# Nuclear tRNA export in trypanosomes: a journey full of twists and turns guided by tRNA modifications

**DOI:** 10.1017/S0031182021000482

**Published:** 2021-09

**Authors:** Zdeněk Paris

**Affiliations:** 1 Institute of Parasitology, Biology Centre, Czech Academy of Sciences, České Budějovice (Budweiss), Czech Republic; 2 Faculty of Science, University of South Bohemia, České Budějovice (Budweiss), Czech Republic

**Keywords:** Nuclear tRNA export, *Trypanosoma brucei*, tRNA modification

## Abstract

Transfer RNAs play a key role in protein synthesis. Following transcription, tRNAs are extensively processed prior to their departure from the nucleus to become fully functional during translation. This includes removal of 5′ leaders and 3′ trailers by a specific endo- and/or exonuclease, 3′ CCA tail addition, posttranscriptional modifications and in some cases intron removal. In this minireview, the critical factors of nuclear tRNA trafficking are described based on studies in classical models such as yeast and human cell lines. In addition, recent findings and identification of novel regulatory loops of nuclear tRNA trafficking in trypanosomes are discussed with emphasis on tRNA modifications. The comparison between the representatives of opisthokonts and excavates serves here to understand the evolutionary conservation and diversity of nuclear tRNA export mechanisms.

## Graphical abstract

**Figure:**
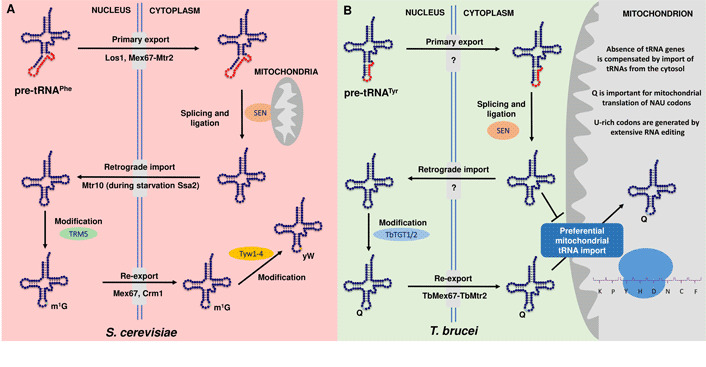

## Introduction

Although tRNAs are smaller than the 40 kDa limit for passive diffusion, the export of tRNAs through the nuclear pore complex (NPC) is an active process facilitated by export factors belonging to the karyopherin-*β* family called exportins. It is now well accepted that tRNA subcellular trafficking is not unidirectional from the site of transcription in the nucleus to the cytoplasmic site of protein synthesis. Interestingly, tRNAs can traffic from the cytoplasm back to the nucleus *via* the tRNA retrograde pathway and then again be re-exported to the cytoplasm (for a recent review, see Hopper and Nostramo, 2019). The tRNA retrograde pathway was discovered in yeast, where intron-containing tRNAs travel across the nuclear pore complex to the outer mitochondrial surface where the splicing endonuclease complex is localized (Yoshihisa *et al*., 2003; Shaheen and Hopper, 2005; Takano *et al*., 2005). Once spliced in the cytoplasm, tRNAs travel back to the nucleus to be further modified. They are then re-exported to the cytosol, where they participate in protein synthesis (Yoshihisa *et al*., 2007). This ‘shuttling’ mechanism has been documented in several model organisms, including humans, but its biological significance is poorly understood. However, in the yeast *Saccharomyces cerevisiae*, tRNA retrograde import was proposed as a level of tRNA quality control, which monitors both the end processing and modification state of tRNAs (Kramer and Hopper, 2013).

A key players in the nuclear tRNA export/import are exportins Los1 and Msn5 and their homologs in vertebrates exportin-t (Xpo-t) and exportin 5 (Xpo-5) (Okamura *et al*., 2014). As shown in previous work (Calado *et al*., 2002) and by a recent comprehensive study employing a co-immunoprecipitation approach, these two proteins serve overlapping but distinct roles in tRNA nuclear export (Huang and Hopper, 2015). Los1 interacts with both spliced and unspliced tRNAs, regardless of whether they are aminoacylated or not, implying that Los1 participates in primary nuclear export and re-export of tRNA to the cytosol. Whereas, Msn5 preferentially binds with spliced and aminoacylated tRNAs establishing its role in tRNA nuclear re-export (Huang and Hopper, 2015). In addition, translation elongation factor 1 *α* was identified in a complex with Msn5, possibly providing the specificity of Msn5 for aminoacylated tRNAs (Calado *et al*., 2002; Huang and Hopper, 2015). In contrast, vertebrate Xpo-5 preferentially exports miRNA, and thus its role in tRNA nuclear export is assumed to be minor (Calado *et al*., 2002). Despite the prominent role of Los1 and Msn5 in the translocation of tRNAs across the NPC, they cannot be the only nuclear exporters since double mutants of these two proteins are viable, suggesting additional export pathways exist and remain uncharacterized (Huang and Hopper, 2015). Recently, a genome-wide screen in yeast revealed new possible players in the tRNA nuclear export (Wu *et al*., 2015). This includes proteins described for their function in rRNA, mRNA and protein export. One of these candidates is represented by the heterodimeric complex of Mex67-Mtr2, well characterized for their essential role in mRNA nuclear export. Inactivation of Mex67-Mtr2 leads to a rapid accumulation of end matured unspliced tRNAs in the nucleus supporting their co-function with Los1 in the primary export pathway. Surprisingly, only four out of ten intron-containing tRNAs were retained in the nucleus, which suggests substrate preference (Chatterjee *et al*., 2017).

Nuclear export of tRNAs has been extensively studied in several model systems, yet there are still key factors missing and this essential pathway is not fully understood. Compared to other eukaryotes, kinetoplastid parasites show unusual features in nuclear and organellar gene expression. These include processes such as mitochondrial RNA editing, mitochondrial tRNA import and trans-splicing of polycistronic mRNAs. There is also a general lack of transcriptional promoters; consequently, gene expression in trypanosomatids is controlled mostly by posttranscriptional pathways (Daniels *et al*., 2010). Nucleo-cytoplasmic tRNA pools may change under certain conditions such as nutrient starvation and oxidative stress (Shaheen and Hopper, 2005; Whitney *et al*., 2007; Chafe *et al*., 2011; Dhakal *et al*., 2019; Schwenzer *et al*., 2019). The trafficking of tRNAs between the nucleus and the cytoplasm might be an additional posttranscriptional event involved in gene regulation with a great importance in the complex life cycle, as these parasites have to face completely different environments with distinct sources of nutrients during the transition between the mammalian and insect stages (Fenn and Matthews, 2007).

Whereas mechanisms for rRNA and mRNA transport in these parasites have been described, there is only limited knowledge about tRNA nuclear export (Dostalova *et al*., 2013; Bühlmann *et al*., 2015). Recent findings indicated that similar to other eukaryotes, the canonical nuclear tRNA exporters TbXpo-t and TbXpo-5 are not singularly essential for cell viability in *Trypanosoma brucei* (Hegedűsová *et al*., 2019). Yet, contrary to yeast, downregulation of both exportins did not result in nuclear accumulation of mature tRNAs, nor did it abolish the export of intron-containing tRNA. With the goal to identify an alternative pathway, the general mRNA exporters TbMex67-TbMtr2 were downregulated, which resulted in a significant increase of nuclearly localized tRNAs. However, contrary to yeast, TbMex67 and TbMtr2 accumulated different subsets of tRNAs in the nucleus. While the elimination of TbMtr2 prevented the export of all tRNAs tested (except for the only intron-containing tRNA^Tyr^), the silencing of TbMex67 resulted in nuclear accumulation of tRNAs modified with queuosine (Q). In turn, inhibition of tRNA nuclear export also affected the levels of queuosine tRNA modification (Hegedűsová *et al*., 2019). An overlapping and different role of Mex67 and Mtr2 was also suggested for budding yeast. This is however a different matter, considering that *S. cerevisiae* lacks the gene for Q-tRNA modification enzyme (Nostramo and Hopper, 2020). These data demonstrate the dynamic nature of tRNA trafficking depending on their modification status and *vice versa*.

**Fig. 1.**
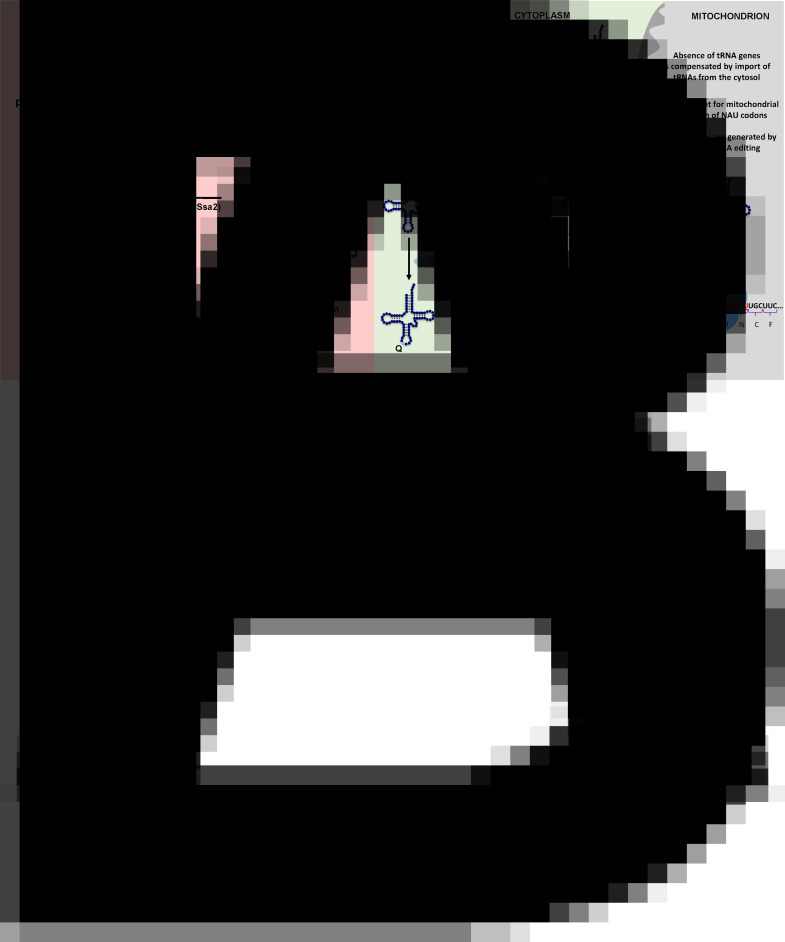
Conservation and diversity of retrograde nuclear trafficking in *S. cerevisiae* and *T. brucei.* **(A)** Model of the retrograde nuclear transport of tRNA^Phe^ in *S. cerevisiae*. Transfer RNAs are synthesized as primary tRNAs (pre-tRNAs) in the nucleus and undergo 5′ and 3′processsing, modifications and CCA addition . In this example, pre-tRNA^Phe^ is subsequently exported from the nucleus to the cytoplasm by Los1 and Mex67-Mtr2 in a step called primary nuclear export. tRNA^Phe^ contains an intron, which is removed by splicing endonuclease (SEN) located at the surface of the mitochondria. Spliced tRNA is then modified by several modification enzymes (not shown) and trafficked back to the nucleus with the help of Mtr10 by a process termed tRNA retrograde import. Ssa2 is also involved in this process only under amino acid starvation. In the nucleus, tRNA^Phe^ is the substrate for the methyltransferase Trm5, which methylates G37 (m^1^G) (in green). In the final step, both Mex67 and Crm1 mediate the constitutive re-export of tRNA^Phe^ to the cytoplasm, where wybutosine (yW) (in yellow) is added to m^1^G in a sequential series of reactions by Tyw1-4. Notably, the canonical exporters Los1 and Msn5 are dispensable in this transport step (Chatterjee *et al*., 2018; Hopper and Nostramo, 2019; Nostramo and Hopper, 2020). **(B)** A model for subcellular trafficking and maturation of the tyrosyl-tRNA (tRNA^Tyr^) in *T. brucei*. The tRNA is transcribed in the nucleus containing an 11-nucleotide long intron. In the nucleus, the intron undergoes non-canonical editing prior to the primary export in the cytoplasm (not shown). Only the edited intron-containing tRNA^Tyr^ is spliced by the SEN complex. After cleavage, tRNA^Tyr^ undergoes retrograde transport to the nucleus to get modified with queuosine (Q) (in blue) by the nuclear enzyme TbTGT1/2. Finally, Q-containing tRNA^Tyr^ is re-exported by TbMex67–TbMtr2 to cytoplasm to serve in cytoplasmic translation. Compared to approximately 50% of Q-containing tRNA^Tyr^ in the cytosol, mitochondria of *T. brucei* contain nearly fully modified tRNA^Tyr^, which could be explained by its preferential import from the cytosol, possibly to play a role in the translation of U-rich tRNAs. The question mark stands for unknown transporter. Note: Except for the only intron-containing (tRNA^Tyr^), TbMtr2 serves as a general exporter in the primary tRNA export, while TbMex67 is responsible for the nuclear export of Q-modified tRNAs (Kessler *et al*., 2017; Hegedűsová *et al*., 2019; Kulkarni *et al*., 2021, under revision in NAR).

Along these lines, in yeast, the process of the retrograde transport pathway proved necessary for 1-methylguanosine (m^1^G) formation at position 37 of tRNA^Phe^, a first step in the synthesis of the hypermodified nucleotide wybutosine (yW). This is because the first step of yW is catalysed by Trm5 methyltransferase that acts only on spliced tRNAs and has a nuclear localization (Ohira and Suzuki, 2011). Consequently, tRNA^Phe^ must be first exported to the cytoplasm by the primary nuclear export pathway to be spliced on the surface of mitochondria, where the tRNA splicing endonuclease is tethered (Fig. 1A). After intron removal, tRNA travels back to the nucleus with the help of the protein Mtr10 to get m^1^G37 and finally is re-exported to the cytoplasm where the remaining four enzymes (Tyw1-4) for wybutosine biosynthesis reside (Ohira and Suzuki, 2011; Nostramo and Hopper, 2020).

An analogous pathway affecting queuosine (Q) modification of the anticodon of tRNA^Tyr^ in *T. brucei* was reported (Kessler *et al*., 2017) (Fig. 1B), where like in yeast, tRNA splicing occurs in the cytoplasm (Yoshihisa *et al*., 2003). Notably, the tRNA-guanine transglycosylase (TGT), the modification enzyme responsible for Q-tRNA formation, resides in the nucleus and it is not able to add Q to an intron-containing tRNA. Therefore, after transcription, processing of the 5′ and 3′ ends and non-canonical intron editing (Rubio *et al*., 2013), the intron-containing tRNA^Tyr^ is exported from the nucleus to be spliced in the cytoplasm by SEN (complex of tRNA splicing endonuclease). After ligation of both exons, tRNA^Tyr^ is imported back to the nucleus to obtain Q and subsequently re-exported to the cytoplasm to fulfil its function in protein synthesis (Kessler *et al*., 2017; Kulkarni *et al*., 2021, under revision in NAR).

However, the situation in *T. brucei* is even more complicated given the fact that the mitochondrial genome is entirely devoid of tRNA genes and all tRNA molecules in the cell have a nuclear origin (Tan *et al*., 2002). Surprisingly, compared to approximately 50% of Q-containing tRNA^Tyr^ in the cytosol, the level of Q modification in the mitochondria is almost 100%, which could be justified by the preferential import of Q-tRNAs and also their ability to translate the mitochondrial predominantly U-rich mRNAs resulting from U-insertion editing (Kulkarni *et al*., unpublished manuscript, under revision in NAR) (Fig. 1B). To elucidate the mechanism of mitochondrial tRNA import, a recent study revealed that tRNAs and proteins may use the same import pathway across the mitochondrial outer membrane but it seems that these two import pathways are not linked (Niemann *et al*., 2017). Still the factors involved in preferential import of Q-modified tRNAs remain to be identified. In addition, the role of tRNA fragments in translation modulation and/or a shortening of the bulk of cellular tRNAs as a result of nutritional stress was recently reported in vertebrate cells as well as in trypanosomes (Fricker *et al*., 2019; Schwenzer *et al*., 2019; Cristodero *et al*., 2021).

Clearly, the complex tRNA biology of kinetoplastid parasites has the potential to provide additional control steps of regulation of gene expression. In conclusion, *T. brucei* provides an ideal model to study the crosstalk between the tRNA trafficking and modification. Nevertheless, further studies based on differences in tRNA pools after silencing of the export factors or facing different nutritional and stress environment together with more complex analyses such as ribosome profiling could identify potential regulatory loops important for the complex lifecycle of these parasites.
